# Prion-like domains as epigenetic regulators, scaffolds for subcellular organization, and drivers of neurodegenerative disease

**DOI:** 10.1016/j.brainres.2016.02.037

**Published:** 2016-03-18

**Authors:** Zachary M. March, Oliver D. King, James Shorter

**Affiliations:** a Department of Biochemistry and Biophysics, Perelman School of Medicine at the University of Pennsylvania, Philadelphia, PA 19104, United States of America; b Biochemistry and Molecular Biophysics Graduate Group, Perelman School of Medicine at the University of Pennsylvania, Philadelphia, PA 19104, United States of America; c Department of Cell and Developmental Biology, University of Massachusetts Medical School, Worcester, MA 01655, United States of America

**Keywords:** RNA-binding proteins, Prion-like domains, Prion, ALS, Disaggregase, Phase transition

## Abstract

Key challenges faced by all cells include how to spatiotemporally organize complex biochemistry and how to respond to environmental fluctuations. The budding yeast *Saccharomyces cerevisiae* harnesses alternative protein folding mediated by yeast prion domains (PrDs) for rapid evolution of new traits in response to environmental stress. Increasingly, it is appreciated that low complexity domains similar in amino acid composition to yeast PrDs (prion-like domains; PrLDs) found in metazoa have a prominent role in subcellular cytoplasmic organization, especially in relation to RNA homeostasis. In this review, we highlight recent advances in our understanding of the role of prions in enabling rapid adaptation to environmental stress in yeast. We also present the complete list of human proteins with PrLDs and discuss the prevalence of the PrLD in nucleic-acid binding proteins that are often connected to neurodegenerative disease, including: ataxin 1, ataxin 2, FUS, TDP-43, TAF15, EWSR1, hnRNPA1, and hnRNPA2. Recent paradigm-shifting advances establish that PrLDs undergo phase transitions to liquid states, which contribute to the structure and biophysics of diverse membraneless organelles. This structural functionality of PrLDs, however, simultaneously increases their propensity for deleterious protein-misfolding events that drive neurodegenerative disease. We suggest that even these PrLD-misfolding events are not irreversible and can be mitigated by natural or engineered protein disaggregases, which could have important therapeutic applications.

## 1. Prions as epigenetic regulators in yeast

Alternative protein folding underpins prion-based phenomena (Lindquist, 1997; Prusiner, 1998; Shorter, 2010). Prions are infectious proteins that can adopt many functionally distinct conformations, at least one of which is self-replicating (Prusiner, 1998; Shorter, 2010). Whereas the mammalian prion protein (PrP) forms prions that cause devastating neurodegenerative diseases (Aguzzi and Lakkaraju, 2016; Colby and Prusiner, 2011; Collinge and Clarke, 2007; Ma and Wang, 2014), prions in yeast are often benign and can even confer selective advantages (Liebman and Chernoff, 2012; Lindquist, 1997; Newby and Lindquist, 2013; Shorter and Lindquist, 2005; Suzuki et al., 2012; True and Lindquist, 2000). Amyloid fibril formation is a unifying feature of both mammalian and fungal prion phenomena, and indeed underpins several age-related neurodegenerative diseases including Alzheimer’s disease and Parkinson’s disease (Colby and Prusiner, 2011; Cushman et al., 2010; Guo and Lee, 2014; Shorter and Lindquist, 2005). Thus, understanding key determinants of this basic biophysical process as it relates to yeast prions will help illuminate new therapeutic strategies for human disease.

The yeast prion states, [*PSI*^+^] and [URE3], are embodied by self-replicating conformers of their protein determinants Sup35 and Ure2, respectively (Cox, 1965; Tuite and Serio, 2010; Tuite et al., 2015; Wickner, 1994). Sup35 is a translation termination factor in yeast; the yeast prion state [*PSI*^+^] is associated with a nonsense suppression phenotype (Tuite et al., 2015). Prion-switching behavior of Sup35 can be readily assessed in yeast harboring a premature stop codon in their *ADE1* gene: [*psi*~] cells (which lack Sup35 prions) are red on rich media and require adenine whereas [*PSI*^+^] cells appear white on rich media and can grow on media lacking adenine (Tuite et al., 2015).

Yeast prion formation is typically mediated by modular and transferable prion domains (PrDs), which are low complexity sequences enriched in polar, uncharged amino acids such as glutamine, asparagine, tyrosine, and serine as well as glycine (Alberti et al., 2009; An et al., 2016; Edskes et al., 1999; Li and Lindquist, 2000; Michelitsch and Weissman, 2000; Santoso et al., 2000; Sondheimer and Lindquist, 2000). An exception is found in Mod5, which forms beneficial [*MOD*^+^] prions without a canonical PrD, and instead harbors an amyloid core-forming region enriched in hydrophobic residues (Suzuki and Tanaka, 2013). Importantly, deletion of the PrD from Sup35 and Ure2 does not affect normal protein function (Coschigano and Magasanik, 1991; Ter-Avanesyan et al., 1993). Conversely, PrDs can be appended to model proteins to generate novel engineered prions (Alberti et al., 2009; Li and Lindquist, 2000; Osherovich and Weissman, 2001; Tyedmers et al., 2010). Furthermore, the sequence of the PrD can be scrambled and still encode prion behavior (Ross et al., 2004, 2005). Thus, it is the amino acid composition and not the exact primary sequence of a PrD, which is critical for prionogenesis (Ross et al., 2004, 2005).

The modular, transferable, and randomizable nature of PrDs has proven enormously instructive in the quest to discover novel prions. A hidden Markov model, based on the amino acid composition of PrDs of Sup35, Ure2, and Rnq1, was developed to query the *S. cerevisiae* genome for novel prions (Alberti et al., 2009), yielding ~200 candidates (cPrDs). To characterize these, chimeras consisting of cPrDs fused to the C-terminal domain of Sup35 were expressed in yeast and cPrDs were assessed based on their ability to mimic the [*PSI*^+^] phenotype (e.g. red-white colony color switching) (Alberti et al., 2009). Further refinement of cPrDs by biochemical and yeast phenotypic analysis led to the discovery of Mot3 prions (Alberti et al., 2009; Holmes et al., 2013). Importantly, not all cPrDs conferred prion behavior (Alberti et al., 2009), and subsequent algorithms have been used as filters to improve prediction accuracy and even to design PrDs capable of bona fide prionogenesis (Paul et al., 2015; Toombs et al., 2010, 2012).

[*MOT3*^+^] is a prion state formed by self-replicating con-formers of its protein determinant, Mot3 (Alberti et al., 2009; Halfmann et al., 2012). Mot3 is a transcription factor that modulates a variety of processes including mating, carbon metabolism, and stress response by repressing anaerobic genes such as *DAN1* during aerobic growth (Grishin et al., 1998). Perhaps most importantly, [*MOT3*^+^] governs the acquisition of multicellular growth phenotypes in yeast through transcriptional regulation of *FLO11* (Holmes et al., 2013). [*MOT3*^+^] enables acquisition of facultative multicellular growth phenotypes including invasive growth on poor nitrogen sources, complex colony morphology as a starvation response, and flocculation in liquid media (Holmes et al., 2013). Furthermore, similar results were found in wild strains harboring [*MOT3*^+^] (Halfmann et al., 2012). These findings illustrate the potential of prions to facilitate rapid adaptation to environmental cues (Lindquist, 1997; Shorter and Lindquist, 2005).

Yeast prions can confer selective advantages in various circumstances, but can also be neutral or detrimental in other settings (Du et al., 2015; Halfmann et al., 2012; Holmes et al., 2013; Newby and Lindquist, 2013; Shorter and Lindquist, 2005; Suzuki et al., 2012; Wickner et al., 2011). The beneficial phenotypes conferred by yeast prions are often observed under stress conditions, which has led to the suggestion that yeast prions constitute bet-hedging devices, which can reveal potentially adaptive genetic diversity in fluctuating environments (Du et al., 2015; Garcia and Jarosz, 2014; Halfmann et al., 2010; Halfmann and Lindquist, 2010; Masel and Bergman, 2003; Newby and Lindquist, 2013; Tyedmers et al., 2008). This process is facilitated by the conformational range of PrDs, which can access multiple, distinct cross-β structures or strains (Shorter, 2010). Moreover, protein folding is exquisitely sensitive to environment, allowing even subtle changes to favor one conformation, and therefore one function, over another (Halfmann et al., 2010, 2012; Halfmann and Lindquist, 2010; Holmes et al., 2013). In the case of Sup35 prions, [*PSI^+^*]-mediated stop codon read-through allows expression of cryptic genetic variation that accumulates in 3′ untranslated regions at many genetic loci (Baudin-Baillieu et al., 2014; Halfmann et al., 2012; Namy et al., 2008; True and Lindquist, 2000; True et al., 2004). Indeed, Sup35 prions act as evolutionary capacitors that release cryptic genetic variation under stress to facilitate the rapid evolution of adaptive traits (Masel and Siegal, 2009; Shorter and Lindquist, 2005).

The adaptive significance of yeast prions, particularly Sup35 prions, has been contested (McGlinchey et al., 2011; Nakayashiki et al., 2005; Wickner et al., 2011, 2015). There has been a lack of evidence that Sup35 and Ure2 prions arise in wild yeast (although Rnq1 prions were readily found) (Chernoff et al., 2000; Resende et al., 2003), leading to speculation that prions were merely ‘diseases’ or artifacts of laboratory cultivation (Wickner et al., 2011, 2015). However, a survey of 690 wild yeast strains from diverse biological niches found 10 strains harboring Sup35 prions, 43 harboring Rnq1 prions, and 6 harboring Mot3 prions (Halfmann et al., 2012). Prions conferred a range of phenotypes that increased fitness of these yeast strains under a wide variety of stresses, and the prion phenotypes could become genetically fixed, thus fulfilling key predictions for bet-hedging prions (Halfmann et al., 2012). However, several of the advantageous [*PSI*^+^]-dependent phenotypes of wild yeast strains were not replicated in another study (Wickner et al., 2015). Nonetheless, the preponderance of evidence suggests that yeast prion proteins undergo environmentally-sensitive, alternative folding to effect epigenetic changes that increase fitness in response to fluctuating environments (Garcia and Jarosz, 2014; Halfmann et al., 2012; Newby and Lindquist, 2013; Suzuki et al., 2012).

## 2. Prion-like domains in humans

**Fig. 1:**
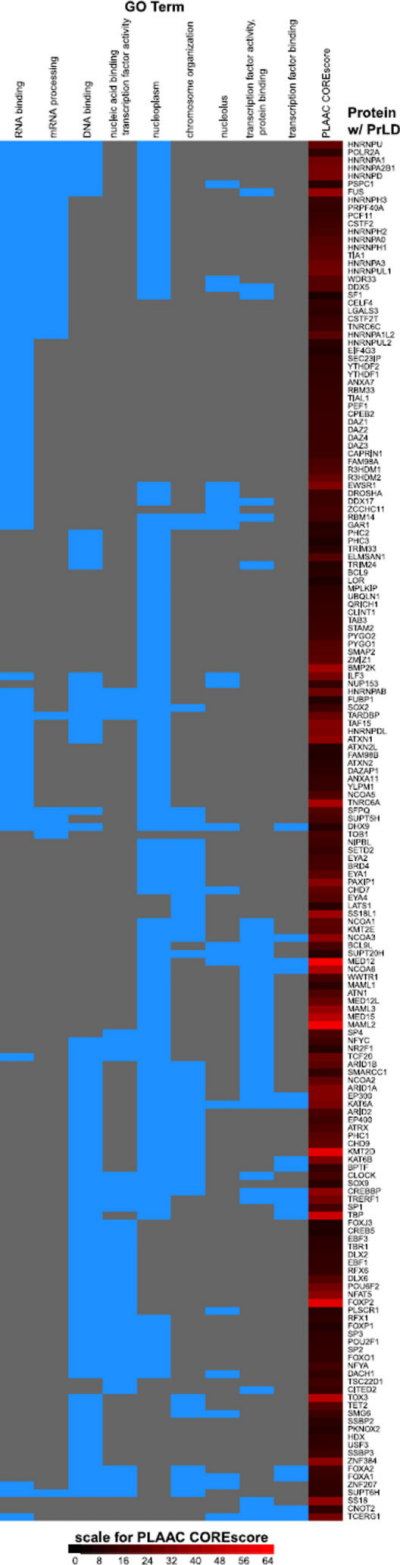
Associations between human PrLD-containing proteins and the Gene Ontology categories enriched for them. Columns correspond to the nine GO Slim categories enriched for human PrLD-containing proteins (Table 1), and rows correspond to the PrLD-containing proteins; the cell indexed by a given row and column is colored blue if the corresponding protein is annotated as belonging to the corresponding category, and gray otherwise. The rows and columns are hierarchically clustered based on correlation of GO Slim annotations. The PLAAC COREscore is also indicated in the far right column using a red color gradient with ranging from black (score 0) to saturated red (score 64), as indicated by the color bar. Rows and columns are ordered by a correlation-based clustering. The 66 of 240 PrLD-containing proteins not associated with any of these categories are not shown (the full list of human proteins with PrLDs is presented in Table S1).

Do human proteins contain domains similar in amino acid composition to yeast PrDs? We applied an updated PrD detection algorithm, PLAAC (for Prion-Like Amino Acid Composition, with default core length of 60 and alpha set to 0.5) (Lancaster et al., 2014), to the human genome (Ensembl GRCh38.p5, release 83) and uncovered 240 genes out of ~20,000 protein-coding genes (~1.2%) harboring a domain compositionally similar to annotated yeast PrDs, termed a prion-like domain (PrLD) (Couthouis et al., 2011; Cushman et al., 2010; Kim et al., 2013; King et al., 2012; Lancaster et al., 2014; Li et al., 2013). The complete list of human proteins with a PrLD, including the location of each PrLD, is presented in Table S1. Remarkably, 72/240 (30%) of these proteins are annotated with the gene ontology (GO) molecular function “RNA binding” and 79/240 (~33%) with the GO molecular function “DNA binding” (Table 1). These are among the nine terms from the generic GO slim (an abridged versions of the full gene ontology) that are significantly enriched for human PrLD-containing proteins (Fisher’s exact test, with Holm’s adjusted *p*<0.05); the others are molecular functions “transcription factor activity, protein binding,” “nucleic acid binding transcription factor activity,” and “transcription factor binding”; biological processes “chromosome organization”, “mRNA processing,” and cellular components “nucleoplasm” and ‘nucleolus’ (Table 1); 174/240 (~73%) of human PrLD-containing proteins were annotated in at least one of these categories (Fig. 1). There are also three GO Slim categories in which PrLD-containing proteins are significantly underrepresented: molecular functions “signal transducer activity” and cellular components “plasma membrane” and “mitochondrion” (Table 1). Our findings suggest that RNA-binding proteins (RBPs) and DNA-binding proteins that reside primarily in the nucleus are significantly overrepresented among the collection of PrLD-containing human proteins (King et al., 2012; Li et al., 2013). Thus, PrLDs feature prominently at the critical functional interfaces between nucleic acid and protein.

Several human RBPs with PrLDs including ataxin 1, ataxin 2, TDP-43, FUS, TAF15, EWSR1, hnRNPA1, and hnRNPA2 (Table S1) feature prominently in the pathology and genetics of a number of fatal neurodegenerative diseases, including amyotrophic lateral sclerosis (ALS), frontotemporal dementia (FTD), and spinocereballar ataxias (Couthouis et al., 2011, 2012, 2014; Cushman et al., 2010; Elden et al., 2010; Kim et al., 2013; Kwiatkowski et al., 2009; Neumann et al., 2006; Orr and Zoghbi, 2007; Orr, 2012; Vance et al., 2009; Zoghbi and Orr, 2009). For example, TDP-43 mislocalizes from the nucleus to cytoplasmic inclusions in degenerating neurons in ALS and FTD, and is an intrinsically aggregation prone protein (Johnson et al., 2009; Ling et al., 2013; Neumann et al., 2006). The PrLD of TDP-43 confers this intrinsically aggregation-prone property (Johnson et al., 2009). Almost all ALS- and FTD-linked TDP-43 mutations lie in the PrLD, and several of these mutations can promote deleterious TDP-43 misfolding and enhance proteotoxicity in diverse model systems (Barmada et al., 2010; Guo et al., 2011; Johnson et al., 2009; Kabashi et al., 2010; Li et al., 2010; Lim et al., 2016; Ling et al., 2013; Ritson et al., 2010; Sreedharan et al., 2008; Zhang et al., 2009). Likewise, multisystem proteinopathy (MSP) can be caused by missense mutations in the PrLD of hnRNPA1 or hnRNPA2 (Kim et al., 2013; Shorter and Taylor, 2013). These mutations alter a gatekeeper aspartate residue and introduce a potent steric zipper motif into the PrLD, which accelerates formation of self-templating hnRNPA1 and hnRNPA2 fibrils (Kim et al., 2013; Shorter and Taylor, 2013). Furthermore, polyglutamine expansions in the PrLD of ataxin 1 cause spinocereballar ataxia 1, and promote ataxin 1 aggregation (Banfi et al., 1994; Cummings et al., 1998). These three striking examples and many others (Chesi et al., 2013; Couthouis et al., 2011, 2012, 2014; Hackman et al., 2013; Klar et al., 2013; Mori et al., 2013; Patel et al., 2015; Vieira et al., 2014) suggest that human proteins with PrLDs are prone to deleterious misfolding events that underpin neurodegenerative disease. Thus, special attention to these proteins is urgently warranted. We suggest that human proteins bearing a PrLD (Table 1) should be scrutinized as potential etiological agents of various degenerative diseases, which might be revealed via gene sequencing and histopathological examination of protein localization (King et al., 2012).

Do human proteins with PrLDs form bona fide prions? Currently, there is no evidence that any human protein with a PrLD can form a prion like PrP, which can naturally transmit devastating neurodegenerative disease between individuals (Colby and Prusiner, 2011; Collinge, 1999; Prusiner, 1998). Nonetheless, PrLDs do enable proteins to spontaneously form self-templating fibrils in isolation (Kim et al., 2013). Intriguingly, ataxin 1 bearing a polyQ expansion within the PrLD can form oligomeric structures that induce local spread of ataxin 1 pathology in transgenic mice (Lasagna-Reeves et al., 2015). Moreover, TDP-43 and TDP-43 fragments containing the PrLD (193–414) can form fibrils that elicit TDP-43 aggregation in cell culture (Furukawa et al., 2011). Furthermore, detergent-insoluble fractions from ALS brains contain TDP-43 fibrils and induce TDP-43 aggregation in cell culture (Nonaka et al., 2013). Thus, TDP-43 may access a prion-like conformation, which may even be transmitted across axon terminals (Feiler et al., 2015). Indeed, phosphorylated TDP-43 pathology in ALS has been interpreted to spread in a sequential manner with highly discernible stages that might indicate involvement of axonal pathways (Brettschneider et al., 2013, 2014; Ludolph and Brettschneider, 2015). Prion-like conformers have been proposed to underlie this spreading phenomenon in ALS and other disorders (Cushman et al., 2010; Grad et al., 2015; King et al., 2012; Li et al., 2013; Maniecka and Polymenidou, 2015; Polymenidou and Cleveland, 2011, 2012; Ravits and La Spada, 2009). Although intriguing, compelling proof of formation of prions will require their *de novo* construction from purely synthetic protein and an ability to infect wild-type mice with a neurodegenerative disease, as has been achieved with PrP and α–synuclein (Luk et al., 2012; Wang et al., 2010, 2011a, 2011b). Even then, only PrP has been shown to form prions that can spread disease naturally between individuals in a population (Colby and Prusiner, 2011; Collinge, 1999; Prusiner, 1998). Evidence is currently lacking that α–synuclein conformers can be infectious in this way.

These various connections with neurodegenerative disease have led to a negative view of PrLDs in human proteins (Cushman et al., 2010; Gitler and Shorter, 2011; King et al., 2012; Li et al., 2013). However, PrLDs are found in 240 human proteins, and so presumably may serve some beneficial or essential function. Indeed, many genes that encode proteins containing PrLDs are essential in mammals (Kraemer et al., 2010; Sephton et al., 2010; Wang et al., 2015). Furthermore, unlike the PrDs of Sup35 and Ure2, the PrLDs of several human proteins, including TDP-43, hnRNPA1, and hnRNPA2 play a critical role in protein function (Li et al., 2013). For example, the PrLD of TDP-43 is not required for RNA- or DNA-binding activity, but is critical for alternative splicing of some mRNAs and for protein-protein interactions with other hnRNPs, including hnRNPA1, hnRNPA2, and FUS, as well components of the Dicer and Drosha complexes (Ayala et al., 2005; Buratti et al., 2005; D’Ambrogio et al., 2009; Kawahara and Mieda-Sato, 2012; Kim et al., 2010). Likewise, the PrLDs of hnRNPA1 and hnRNPA2 make important contributions to the splicing activity of these proteins (Mayeda et al., 1994). Recent reports suggest that PrLDs may have an important role in critical phase transition events that provide organizational scaffolds for various membraneless organelles, including RNP granules and nuclear subcompartments (Brangwynne et al., 2015; Courchaine and Neugebauer, 2015; Guo and Shorter, 2015; Hennig et al., 2015; Kawaguchi and Hirose, 2015; Li et al., 2013; Ramaswami et al., 2013).

## 3. Structure and function of membraneless cellular compartments

In addition to classical membrane-delimited organelles, the eukaryotic cell is also organized by membraneless organelles. These include nucleoli, Cajal bodies, gems, paraspeckles, and PML bodies in the nucleus and processing (P) bodies, stress granules, and P granules in the cytoplasm (Zhu and Brangwynne, 2015). Yet, many questions remain about the physical basis by which these compartments form and function. One hypothesis that has gained significant attention is that these membraneless organelles form through phase separation (Brangwynne et al., 2009; Brangwynne, 2013; Hyman et al., 2014; Li et al., 2012). However, precisely what phase architecture these membraneless organelles adopt has remained the subject of intense scrutiny.

Early evidence that membraneless organelles may be liquid-like came from the study of P granules. In the *Caenorhabditis elegans* embryo, polarization along the anterior-posterior axis leads to accumulation of P granules in the embryo posterior to mark germ cells (Seydoux and Braun, 2006; Strome and Lehmann, 2007). Three-dimensional tracking of fluorescently labeled P granule components revealed that upon symmetry breaking, P granules form spontaneously in the embryo posterior in the vicinity of polarity proteins. Thus, P granule formation is driven by local decreases in the saturating concentration of P granule components in the embryo posterior and an increased flux of P granule components into the embryo posterior (Brangwynne et al., 2009). Furthermore, P granules exhibit classic liquid properties such as fusion, dripping and wetting (Brangwynne et al., 2009, 2015). Together, these data strongly argued for liquid-liquid phase separation as a mechanism for subcellular cytoplasmic architecture.

A small molecule screen for compounds to promote neuronal progenitor cell differentiation into mature neurons led to the serendipitous discovery that biotinylated 5-aryl-isoxazole-3-carboxyamide (b-isox) selectively precipitates known protein components of ribonucleoprotein (RNP) granules, including TDP-43 and FUS (Kato et al., 2012). *In vitro*, recombinant full-length FUS phase transitions to a hydrogel-like state, but very high protein concentrations are required (Kato et al., 2012; Patel et al., 2015). Furthermore, it was observed that hydrogels formed by the PrLD of FUS were capable of retaining soluble FUS PrLD, suggesting that hydro-gel assembly is mediated by homotypic interactions in the FUS PrLD (Kato et al., 2012). Electron microscopy of FUS PrLD hydrogels revealed a composition of amyloid-like fibrils (Kato et al., 2012). X-ray diffraction analysis showed prominent reflections at 4.6–4.7 Å and 10 Å typical of cross-β structure common to amyloid fibrils, again strongly suggesting an amyloid-like structural basis to hydrogel architecture (Kato et al., 2012). These hydrogels and fibrils were readily dissolved upon exposure to SDS or mild (37 °C) heating, suggesting that they were distinct and more dynamic than the SDS-resistant amyloid fibrils formed by yeast prions (Kato et al., 2012; Kryndushkin et al., 2003; Serio et al., 2000; Shorter and Lindquist, 2006). This finding was consistent with previous work demonstrating that purified FUS spontaneously assembles into SDS-soluble fibrils (Sun et al., 2011). However, whether the hydrogels formed *in vitro* were reflective of RNP granules found in cells remained uncertain (Weber and Brangwynne, 2012).

Multiple reports have suggested that RNA granules exist as predominantly liquid compartments and not gel-like compartments in cells (Burke et al., 2015; Lin et al., 2015; Molliex et al., 2015; Murakami et al., 2015; Patel et al., 2015; Zhang et al., 2015). For example, in cells and *in vitro*, FUS forms dynamic assemblies that are rapidly recruited to sites of DNA damage, and display physical characteristics of liquid droplets as predicted by classical physics of polymeric phase transitions, including fast internal dynamics, spherical morphology, and a propensity for two droplets to readily fuse when in close contact with one another (Altmeyer et al., 2015; Burke et al., 2015; Lin et al., 2015; Murakami et al., 2015; Patel et al., 2015). Importantly, the PrLD mediates the phase transition as deletion of the PrLD abrogates droplet assembly. Detailed study of RBPs hnRNPA1, Lsm4, Tia1, and Pub1 revealed similar behavior (Burke et al., 2015; Lin et al., 2015; Molliex et al., 2015). Droplet formation was influenced by the presence of molecular crowding agents, ionic strength, and presence of RNA or other polyanions, such as poly-ADP ribose (Burke et al., 2015; Lin et al., 2015; Molliex et al., 2015; Murakami et al., 2015; Patel et al., 2015; Zhang et al., 2015).

The identity of the bound RNA tunes the biophysical properties of the RNP granule (Zhang et al., 2015). In the filamentous fungus *Ashbya gossypii*, the RBP Whi3 possesses a PrLD that enables assembly into liquid droplets to organize cyclin transcripts (*CLN3*) at sites of nuclear division and formin transcripts (*BNI1*) at polarity sites where new branch sites are located (Zhang et al., 2015). Microrheology studies revealed that Whi3 droplets bound to BNI1 are less viscous than *CLN3*-bound droplets (Zhang et al., 2015). Additionally, BNI1 droplets fuse with one another faster than *CLN3* droplets (Zhang et al., 2015). This suggests that client RNA identity is critical to tuning the biophysical properties of RNP granules, and that different physical properties of RNP granules may be optimized for specific cellular functions (Guo and Shorter, 2015; Zhang et al., 2015). This latter point raises a provocative parallel with yeast prions, where conformational diversity gives rise to distinct prion strains with unique phenotypes (King and Diaz-Avalos, 2004; Roberts et al., 2009; Shorter, 2010; Tanaka et al., 2004). It is possible that bound RNAs may define ‘strains’ of RNP granules by tuning their biophysical properties.

The case that biophysical properties of RNP granules reflect their functional role is perhaps most strikingly made in *S. cerevisiae*. Specifically, whereas P bodies are constitutively active sites involved in mRNA processing and degradation and exist as liquid droplets in yeast, stress granules are inactive storage sites for proteins and RNA that form rapidly upon onset of stress, and are solid, gel-like aggregates (Balagopal and Parker, 2009; Kroschwald et al., 2015). Yeast rely on the protein disaggregase and hexameric AAA+ ATPase, Hsp104 (DeSantis and Shorter, 2012), to maintain the fluidity of P bodies (Kroschwald et al., 2015). Metazoa lack an Hsp104 homolog (Erives and Fassler, 2015; Shorter, 2008), and curiously in mammalian cells P bodies and stress granules exist in more liquid-like states (Kroschwald et al., 2015). Thus, the powerful disaggregase activity of Hsp104 may enable yeast cells to readily exploit solid, gel, and liquid states in RNP granules (Kroschwald et al., 2015).

What structure(s) do the PrLDs of RNA-binding proteins adopt in liquid droplets? Solution NMR study of the FUS PrLD in monodisperse solution and condensed into droplets revealed that the PrLD retains disordered character in droplets (Burke et al., 2015). This finding suggests a model in which these liquid droplets maintain rapid internal dynamics while being held together by transient intermolecular contacts between adjacent PrLDs (Burke et al., 2015).

In an attempt to further address this question, a mass spectrometry-based chemical footprinting method has been employed in which N-acetylimidazole (NAI) is used to acetylate serine, tyrosine, lysine, threonine, arginine, and asparagine side chains in proteins (Xiang et al., 2015). Using two model proteins, recombinant glutathione-S transferase (GST) and poly-ADP-ribose polymerase (PARP) isolated from HEK 293T cell nuclei, it was shown that solvent-accessible side chains (as assessed by available crystallographic data for these two proteins) are more readily acetylated (Xiang et al., 2015). Thus, the acetylation pattern or “footprint” for a given protein can be used as a conformational proxy (Xiang et al., 2015).

This technique was then deployed to show that the PrLD of hnRNPA2 in droplets or polymerized into hydrogels in vitro, or hnRNPA2 isolated from nuclei adopt similar conformations, as assessed by their NAI footprints (Xiang et al., 2015). This congruence might suggest that cross-β fibrillization underpins both phase transitions to liquid droplets and hydrogel formation (Xiang et al., 2015). Curiously, however, hnRNPA2 PrLD fused to maltose binding protein (MBP) to maintain the hnRNPA2 PrLD in a soluble, monomeric state also displayed a footprint qualitatively similar to that obtained for hydrogels, liquid droplets, and fibrils (Xiang et al., 2015). Therefore, it is difficult to interpret precisely how the hnRNPA2 chemical footprints relate to hnRNPA2 structure. One possibility is that the observed NAI footprint in the monomeric state may be due to contamination by small amounts of fibrils. This possibility is supported by the fact that the intensity of the footprint progressively increased with time after MBP cleavage, and suggests that the NAI footprinting method detects hnRNPA2 fibril abundance. Alternatively, a fraction of hnRNPA2 PrLDs might exhibit cross-β structure even in the context of monomeric, soluble hnRNPA2. Although the hnRNPA2 PrLD is predicted to be intrinsically disordered in the soluble, monomeric state (Kim et al., 2013), circular dichroism studies suggest that this domain may adopt β-sheet-rich structures in solution (Landsberg et al., 2006). Another possibility is that similar regions within the hnRNPA2 PrLD may be invariably solvent accessible (or inaccessible) in distinct structures for monomeric forms in solution, in liquid phases, and in cross-β fibrils, and consequently the NAI footprint does not resolve between them. We suggest that further structural studies using complementary techniques in addition to NAI foot-printing are needed to further resolve the structure of the hnRNPA2 PrLD in various soluble, liquid, and fibrillar states.

Parker and colleagues observed that stress granules contain stable subcompartments that can be isolated in cell lysates, thus suggesting that stress granules are not simply liquids (Jain et al., 2016). Instead, they propose that stress granules contain stable, gel-like cores surrounded by a dynamic liquid shell (Jain et al., 2016). They demonstrate that the cores of stress granules isolated from yeast are larger than their counterparts from mammalian cells, thus reconciling their data with that of Alberti and colleagues (Kroschwald et al., 2015). The existence of a stable, gel-like core within stress granules may even provide an explanation for the observation by McKnight and colleagues that nuclear hnRNPA2 has a similar chemical footprint to recombinant hnRNPA2 polymerized into hydrogels (Xiang et al., 2015). However, it remains unclear whether cross-β polymerization is at the root of the stable stress granule core (Jain et al., 2016; Xiang et al., 2015). Thus, the structure of PrLDs within RNP granules is likely to remain the subject of intense focus in future studies. Moreover, other membraneless organelles, such as the nucleolus, contain distinct subcompartments (Boisvert et al., 2007). It will be important to determine whether these are due to separated gel and liquid phases, or immiscible liquid phases with different viscosities.

## 4. Membraneless organelles and prion-like domains: a mechanistic link between normal physiology and neurodegenerative disease

What is the connection between the functional role of PrLDs in beneficial phase transitions in the formation of membraneless organelles and the deleterious misfolding events that these domains undergo in neurodegenerative disease? Recent work *in vitro* demonstrates that liquid droplets composed of FUS and hnRNPA1 harboring disease-linked mutations in their PrLDs (FUS^G156E^ and hnRNPA1^D262V^) mature to a solid, hydrogel-like state more rapidly than droplets formed by wild-type protein (Molliex et al., 2015; Murakami et al., 2015; Patel et al., 2015). Moreover, hydrogel-like forms of mutant FUS have been specifically associated with neurodegenerative phenotypes in a *C. elegans* model of FUS proteinopathy (Murakami et al., 2015). Thus, a direct link emerges between the biophysical propensity of these proteins to adopt more solid-like structures and neurodegeneration.

Interestingly, the majority of ALS-causing mutations in FUS are found not in the PrLD but in the C-terminal region (Da Cruz and Cleveland, 2011; Kwiatkowski et al., 2009; Vance et al., 2009), where they disrupt a proline-tyrosine (PY) nuclear localization signal (Lee et al., 2006; Suel et al., 2008; Zhang and Chook, 2012). Mutations in the FUS PY-NLS lead to persistent cytoplasmic FUS mislocalization, which correlates with ALS severity (Dormann et al., 2010), but curiously does not directly increase the biophysical propensity of FUS to aggregate (Sun et al., 2011). However, the biophysics of membraneless organelle assembly shed new light on this observation. Membraneless organelles such as nucleoli and P granules in *Caenorhabditis elegans* have the property that local concentration of granular components drive granule droplet condensation (Brangwynne et al., 2015; Weber and Brangwynne, 2015; Zhu and Brangwynne, 2015). Thus, a model emerges where impaired nuclear import of FUS leads to persistent cytoplasmic FUS droplets that mature to more intractable solid aggregates, in accordance with findings in vitro (Lin et al., 2015; Molliex et al., 2015; Murakami et al., 2015; Patel et al., 2015). Therefore, strategies to boost FUS nuclear import or maintain FUS droplet fluidity should represent a significant therapeutic opportunity for ALS and FTD.

## 5. Clearance mechanisms for solid protein aggregates

In *S. cerevisiae*, the protein-remodeling factor Hsp104 regulates the formation, elimination, and propagation of beneficial yeast prions (Sweeny and Shorter, 2008). Hsp104 severs yeast prions to ensure their dissemination to daughter cells upon division (Satpute-Krishnan et al., 2007; Shorter and Lindquist, 2004, 2006, 2008; Sweeny et al., 2015; Sweeny and Shorter, 2015), and is required to clear solid stress granules in yeast and to maintain the fluid integrity of yeast P bodies (Cherkasov et al., 2013; Kroschwald et al., 2015). Indeed, it is hypothesized that this robust disaggregase machinery coevolved with solid stress granules as a way for yeast to cope with their vulnerability to environmental fluctuation (Kroschwald et al., 2015). However, metazoa lack a clear Hsp104 homolog (Erives and Fassler, 2015), and fibrils formed from human RBPs with PrLDs represent an intractable substrate for wild-type Hsp104 (Jackrel et al., 2014). Recently, however, engineered forms of Hsp104 have been generated that potently suppress the aggregation and toxicity of various disease-linked RBPs with PrLDs, including TDP-43, FUS, and TAF15 (Jackrel et al., 2014, 2015; Jackrel and Shorter, 2014a; Sweeny and Shorter, 2015; Torrente et al., 2016). Moreover, these potentiated Hsp104 variants dissolve preformed TDP-43, FUS, and TAF15 fibrils *in vitro* (Jackrel et al., 2014; Jackrel and Shorter, 2014a). Thus, Hsp104 could represent a disruptive technology to enhance metazoan proteostasis to counter RBP misfolding that causes neurodegenerative disease such as ALS and FTD (Jackrel and Shorter, 2014b, 2015).

In metazoan cells and yeast, the Hsp110, Hsp70, and Hsp40 protein-disaggregase machinery (Nillegoda and Bukau, 2015; Shorter, 2011; Torrente and Shorter, 2013), contributes to the clearance of stress granules (Cherkasov et al., 2013; Kroschwald et al., 2015; Walters et al., 2015). Hsp70 and Hsp40 chaperones often get sequestered and inactivated by misfolded protein aggregates (Auluck et al., 2002; Derkatch and Liebman, 2013; Yu et al., 2014). Thus, enhancement or engineering of this disaggregase machinery might also open potential therapeutic avenues for ALS, FTD, and a variety of other neurodegenerative disorders.

Deletion of several autophagy-related genes and Cdc48 adaptor proteins gives rise to constitutive stress granules in *S. cerevisiae* (Buchan et al., 2013). Cdc48, or valosin-containing protein (VCP) in humans, is another hexameric AAAþ ATPase that can promote autophagy (Ju et al., 2009; Krick et al., 2010; Meyer et al., 2012), and mutations in VCP cause familial forms of ALS and MSP (Johnson et al., 2010). These diseases are characterized by the formation of cytoplasmic protein aggregates containing RBP components of stress granules (Li et al., 2013). Deletion of the autophagy gene ATG7 or siRNA-mediated knockdown of VCP leads to impaired ability to clear stress granules in mammalian cells, and disease-causing mutations in VCP cause accumulation of constitutive stress granules that contain TDP-43 (Buchan et al., 2013). However, it remains unclear whether the sole role of Cdc48/VCP is to target stress granule components for autophagic degradation or whether Cdc48/VCP may have also have an active role in disaggregation of stress granules and reactivation of their components (Buchan et al., 2013). Indeed, an exciting possibility is that Cdc48/VCP may represent a triage center for stress granule components, effecting the reactivation of salvageable components and the degradation of others.

## 6. Concluding remarks

**Fig. 2:**
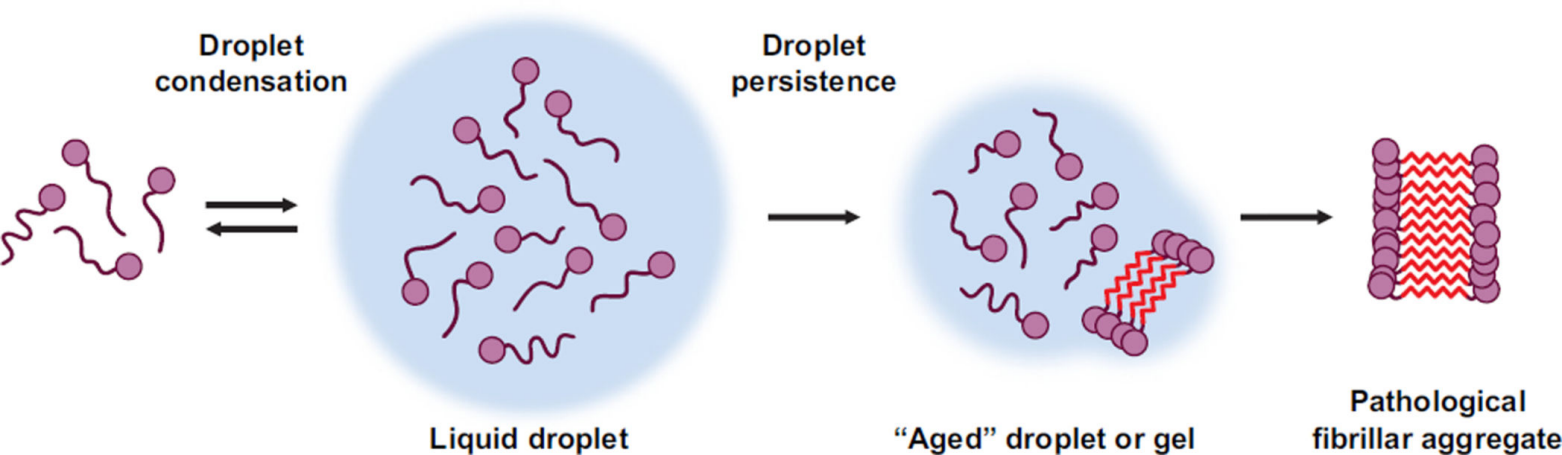
Phase transitions of prion-like domains. RNA-binding proteins (RNA recognition motif depicted by purple circles) can reversibly coalesce into dynamic liquid droplets through transient interactions in their prion-like domains (PrLDs depicted as purple lines). Droplet persistence over time, or mutations in PrLDs that introduce potent steric zippers, can drive further condensation of aged droplets into a less dynamic form that can give rise to solid fibrillar protein aggregates that accrue in neurodegenerative disease.

Here, we have reviewed recent advances in our understanding of prion-like phenomena and architecture across biology, from epigenetic regulation in the simple model organism *S. cerevisiae* to complex mechanisms of eukaryotic subcellular organization. We suggest that PrDs and PrLDs may have been biologically conserved for their wide-ranging biological utility. The ability of prions to rapidly switch between distinct conformational and functional states confers selective advantages for yeast in the face of environmental stress (Halfmann et al., 2012; Holmes et al., 2013; Suzuki et al., 2012; True and Lindquist, 2000; True et al., 2004). In fungi and metazoa, PrLDs now have a clear functional role in mediating the reversible coalescence of RNP granules. However, in humans the persistence and maturation of these RNP granules via complex mechanisms leads to pathological protein accumulation and neurodegenerative disease (Fig. 2) (Guo and Shorter, 2015; Lin et al., 2015; Molliex et al., 2015; Murakami et al., 2015; Patel et al., 2015; Xiang et al., 2015). We suggest that PrLDs may be general scaffolds for membraneless subcellular organization. However, this activity places PrLDs at risk to accessing deleterious misfolding trajectories that cause neurodegenerative disease (Li et al., 2013). PrLDs have a very distinctive amino acid composition, but this role in subcellular compartmentalization via phase transitions and simultaneous risk of protein misfolding may extend to other intrinsically unfolded, low complexity domains with different amino acid composition. Regardless, we suggest that deleterious misfolding events can be reversed by select protein disaggregases, which could have important therapeutic applications (Jackrel and Shorter, 2015; Shorter, 2008; Torrente and Shorter, 2013).

## Supplementary Material

1

## Figures and Tables

**Table 1:** Gene Ontology categories in which human PrLD-containing proteins are significantly over- or under-represented. Fisher’s exact test was used to test for independence between the set of human proteins with PrLDs and GO Slim gene annotations for 129 categories (14 with fewer than 5 or more than 5000 annotated genes were excluded); the categories with *p*-value<0.05 after adjusting for multiple hypothesis testing with Holm’s method are shown. Columns give the GO ID, branch of the ontology, GO term, number of genes with this annotation overall (n.total) and among PrLD containing-proteins (n.PrLD), odds ratio, raw *p*-value, and Holm’s-adjusted *p*-value. Only the longest protein-coding transcript for each HUGO gene name was included in the analyses to avoid redundancies, and 906 genes with no GO Slim annotations (including 4 with PrLDs) were excluded from the gene universe, as were those encoding proteins of fewer than 60 amino acids, leaving 18,672 genes in the universe, 236 of them with PrLDs.

GO.id	GO.branch	GO.term	n.total	n.PrLD	Odds.ratio	p.raw	p.adjusted
Over-represented							
GO:0005654	CC	Nucleoplasm	2879	116	5.48	2.2e–34	2.9e–32
GO:0003723	MF	RNA binding	1545	72	5.05	2.5e–23	3.2e–21
GO:0000988	MF	Transcription factor activity, protein binding	516	37	6.97	8.8e–18	1.1e–15
GO:0003677	MF	DNA binding	2320	79	3.64	2.3e–17	2.9e–15
GO:0006397	BP	mRNA processing	430	31	6.83	4.4e–15	5.4e–13
GO:0051276	BP	Chromosome organization	971	44	4.33	1.1e–13	1.4e–11
GO:0001071	MF	Nucleic acid binding transcription factor activity	1077	39	3.32	2.5e–09	3.1e–07
GO:0008134	MF	Transcription factor binding	432	16	3.15	1.3e–04	1.6e–02
GO:0005730	CC	Nucleolus	810	23	2.42	2.8e–04	3.4e–02
Under-represented							
GO:0005886	CC	Plasma membrane	4539	23	0.33	1.6e–08	1.9e–06
GO:0004871	MF	Signal transducer activity	1566	4	0.19	2.5e–05	3.0e–03
GO:0005739	CC	Mitochondrion	1319	4	0.22	2.8e–04	3.4e–02
